# Theta burst stimulation on the fronto-cerebellar connective network promotes cognitive processing speed in the simple cognitive task

**DOI:** 10.3389/fnhum.2024.1387299

**Published:** 2024-07-19

**Authors:** Ning Zhao, Jing Tao, Clive Wong, Jing-song Wu, Jiao Liu, Li-dian Chen, Tatia M. C. Lee, Yanwen Xu, Chetwyn C. H. Chan

**Affiliations:** ^1^College of Rehabilitation Medicine, Fujian University of Traditional Chinese Medicine, Fuzhou, China; ^2^Department of Rehabilitation, The 6th Affiliated Hospital of Shenzhen University Medical School, Shenzhen, China; ^3^National-Local Joint Engineering Research Center of Rehabilitation Medicine Technology, Fujian University of Traditional Chinese Medicine, Fuzhou, China; ^4^Department of Psychology, The Education University of Hong Kong, Tai Po, Hong Kong SAR, China; ^5^State Key Laboratory of Brain and Cognitive Sciences, The University of Hong Kong, Pokfulam, Hong Kong SAR, China; ^6^Laboratory of Neuropsychology and Human Neuroscience, Department of Psychology, The University of Hong Kong, Pokfulam, Hong Kong SAR, China; ^7^Department of Rehabilitation Medicine, Affiliated Hospital of Soochow University, Wuxi, China

**Keywords:** fronto-cerebellar, functional connectivity, TMS, cognitive processing speed, reaction time

## Abstract

**Background:**

The fronto-cerebellar functional network has been proposed to subserve cognitive processing speed. This study aims to elucidate how the long-range frontal-to-cerebellar effective connectivity contributes to faster speed.

**Methods:**

In total, 60 healthy participants were randomly allocated to three five-daily sessions of transcranial magnetic stimulation conditions, namely intermittent theta-burst stimulation (iTBS, excitatory), continuous theta-burst stimulation (CTBS, inhibitory), or a sham condition. The sites of the stimulations were the right pre-supplementary motor area (RpSMA), medial cerebellar vermis VI (MCV6), and vertex, respectively. Performances in two reaction time tasks were recorded at different time points.

**Results:**

Post-stimulation speeds revealed marginal decreases in the simple but not complex task. Nevertheless, participants in the excitatory RpSMA and inhibitory MCV6 conditions showed direct and negative path effects on faster speeds compared to the sham condition in the simple reaction time (SRT) task (*β =* −0.320, *p =* 0.045 and *β =* −0.414, *p =* 0.007, respectively). These path effects were not observed in the SDMT task.

**Discussion:**

RpSMA and MCV6 were involved in promoting the path effects of faster reaction times on simple cognitive task. This study offers further evidence to support their roles within the long-range frontal-to-cerebellar connectivity subserving cognitive processing speed. The enhancement effects, however, are likely limited to simple rather than complex mental operations.

## 1 Introduction

Cognitive processing speed (PS-C) refers to the time required to encode an incoming stimulus and connect it with the existing experience in the brain (Kail and Salthouse, 1994). Previous fMRI studies revealed that PS-C is subserved by extensive neural networks. Silva et al. (2018) revealed that performance on a symbol-to-digit task was subserved by an extensive network of middle frontal gyri, superior parietal lobule, precuneus, inferior frontal gyrus, cuneus, and lingual gyrus (Silva et al., 2018). Previous studies quantified processing speed with complex cognitive tests, such as the Symbol Digit Modalities Test (SDMT). A review of these studies indicates their results are inconsistent. For instance, Silva et al. (2019) reported the fronto-parietal and fronto-occipital networks subserved participants’ performances in the SDMT (Silva et al., 2019). Savini et al. (2019) revealed that the connectivity between the cerebellum and the default mode network (DMN) was related to the reaction time decline in the SDMT among multiple sclerosis patients (Savini et al., 2019). Manca et al. (2018) conducted a systematic review of multiple sclerosis patients that connectivity of the frontal areas and microstructural integrity of the anterior corpus callosum accounted for processing speed tasks, including SDMT (Manca et al., 2018). The inconsistent results mentioned above could have been confounded by varying contents and task-taking processes of the complex cognitive tasks used in these studies, such as the SDMT.

In a recent study, our research team designed a simple multimodality reaction time task to revisit the PS-C (Wong et al., 2021). This custom-designed task was developed to minimize the potential task-specificity confounds observed in previous studies. The results indicated a fronto-cerebellar connective network, especially long-range effective connectivity in the right medial frontal cortex on the medial cerebellar vermis VI [i.e., Right medial frontal cortex (RMFC) → medial cerebellar vermis VI (MCV6)] subserving PS-C. We further proposed that PS-C is the outcome of an interplay between automaticity and effortful top-down attentional control processes (Wong et al., 2021).

In this study, we aimed to gain a deeper understanding of the roles of the RMFC and MCV6 within the fronto-cerebellar connective network in subserving PS-C. The reason for selecting the RMFC–MCV6 couple for the study is because it was the strongest effective connective predictor of faster reaction times (with the largest *β* value of −0.330) among the six significant paths (Wong et al., 2021). The RMFC–MCV6 couple is one of the three long-range functional connectivities between the frontal cortex and cerebellum, which involve automaticity and top-down attentional control interplay. The changes in task-based reaction times due to external stimulations applied to the RMFC–MCV6 couple would yield stronger modulation of the fronto-cerebellar connective network than other couples that showed weaker prediction power or belonged to short-range such as the RMFC–LIPS (LIPS refers to left intra-parietal sulcus; *β* value of 0.301).

We employed transcranial magnetic stimulation (TMS) to separately modulate the neural activities of the RMFC and MCV6. Previous studies have demonstrated the effects of TMS on regulating brain activation (Burke et al., 2019) and inducing functional and structural plastic changes (Jung and Lambon Ralph, 2021). The intermittent theta-burst stimulation (iTBS) protocol induced excitatory effects, while the continuous theta-burst stimulation protocol (CTBS) induced inhibitory effects. Other literature employed stimulation to a single neural substrate method to modulate the connectivity of neural networks. For instance, intermittent TBS of the left superior parietal lobule was revealed to enhance cognitive speed and resting-state connectivity of the dorsal attention network (DAN) (Anderkova et al., 2018). Intermittent theta-burst stimulation at the midline cerebellar node of the DAN resulted in improved performances in both sustained and transient attentional control functions, which subserved by the respective connective network (Esterman et al., 2017). High-frequency rTMS of the lateral parietal region was found to effectively improve generic cognitive function via activating the default mode network (DMN) (Wei et al., 2022). The significant single substrate-to-network modulation effects reported in the previous studies lend support to our use of such a method in this study.

The RMFC→MCV6 couple connectivity was a negative relationship (denoted by the →) between the two neural substrates (Wong et al., 2021). That is, the RMFC exerted an inhibitory influence on the MCV6. The negative *β* value of −0.330 in the regression model suggested that lowering the inhibitory RMFC→MCV6 influence would produce faster PS-C. First, we aimed to increase the inhibitory influence of the RMFC on the MCV6 by applying an excitatory iTBS protocol to the right pre-supplementary motor area (RpSMA). The RpSMA, a subregion of the RMFC (De La Vega et al., 2016), is located in the right prefrontal cortex as part of the salient network (Wang et al., 2020). The RpSMA was found to be related to resource allocation in processing salient information (Weigard et al., 2019) and cognitive control (Wolpe et al., 2022). A previous study of six sessions of excitatory stimulation over the frontal cortex showed increased cognitive performance in patients with Parkinson’s disease (Trung et al., 2019). Other studies (Viejo-Sobera et al., 2017; Chung et al., 2019; Dumitru et al., 2020; Wu et al., 2021) reported that the excitatory effects promoted general cognitive functions among healthy subjects and modulated cerebellar-cortical connectivity in patients with progressive supranuclear palsy (Brusa et al., 2014). Second, to produce contrasting effects, an inhibitory CTBS protocol was applied to the MCV6 to suppress its activity. The cerebellar vermis is related to automation, cognitive optimization, and implicit learning (Cheng et al., 2014; Moroso et al., 2017) and modulates neural synchronization in the non-motor frontal cortex (Tremblay et al., 2019). Multiple sessions of inhibitory stimulation over the cerebellar vermis have been revealed to promote synaptic connections (Colnaghi et al., 2017a). In contrast to the facilitative effects mentioned above, other studies reported mixed results of the inhibitory stimulation to the cerebellum.

We hypothesized that iTBS to the RpSMA, which is excitatory in nature, would further increase the negative influence on the MCV6, resulting in higher neural activity to mediate faster reaction times in the participants’ post-intervention task performances. In contrast, as the MCV6 receives negative influence from the RMFC (i.e., RMFC→MCV6) to produce faster reaction time, CTBS to the MCV6, which would inhibit its activity, was hypothesized to produce slower reaction times. We employed a two-task contrast method to quantify the post-stimulation effects. They were the simple reaction time task (SRT), vs the SDMT, a complex reaction time task. The TMS effects were analyzed with repeated measure analysis of covariance (RM-ANCOVA) for the Group × Time on the changes in the task-based reaction times. Structural equation modeling (SEM) was used to model the differences in the TMS modulations of the participants’ reaction times on the simple vs complex tasks.

## 2 Materials and methods

### 2.1 Subjects

A total of 60 young adults (men: 20; women: 40) were recruited from the university where the study was carried out and its surrounding communities in Fuzhou, China. The participants were randomly assigned to the iTBS, CTBS, or SHAM groups (*n* = 20 each). Their mean age was 23.08 ± 2.31 years, and their years of education ranged from 16.1 to 16.4. All participants had normal or corrected vision without color blindness and normal hearing function of >40 decibels at 500–4,000 Hz. The participants were all right-handed. The exclusion criteria were as follows: participants (1) with a history of known medical, neurological, and mental disorders; (2) with alcohol and other substance abuse habits; (3) with the use of epileptic and hypertension medication; and (4) who are pregnant. Ethics approval was obtained from the Ethics Committee of the Rehabilitation Hospital affiliated with the Fujian University of Traditional Chinese Medicine (No.2019YJS-003-04). All participants provided informed consent before the intake measurement and participation in the experiment.

Sample size calculation was based on the effect size of 0.723 reported in the study by Rastogi et al. (2017), which applied TMS over the lateral cerebellum vs sham. To achieve a power of 0.95 for three groups and four time points at *α* = 0.05, the sample size was 50 participants. With an attrition rate of 15%, the final sample size was 60 participants.

### 2.2 Study design and experimental setup

**Figure 1:**
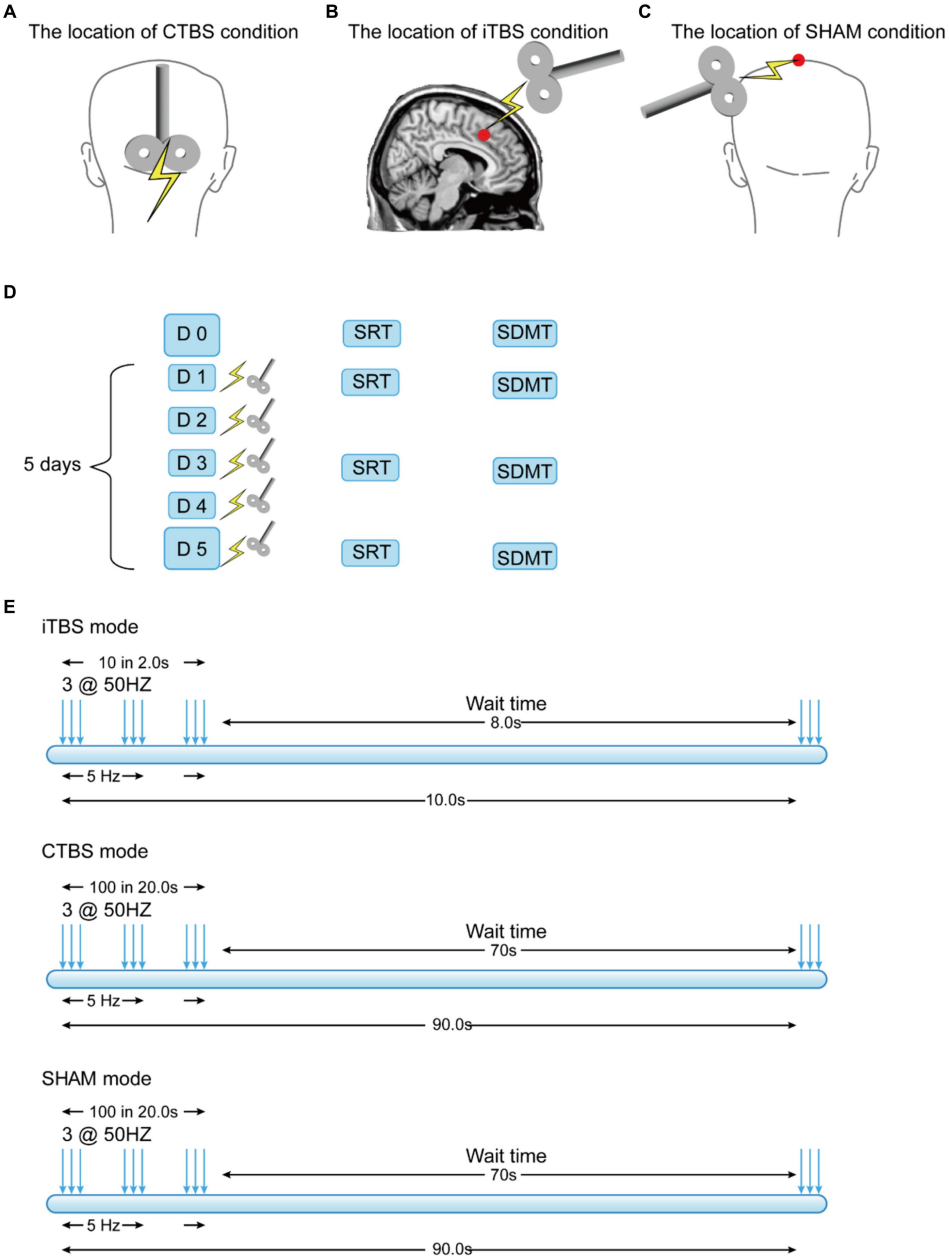
Locations over the participants’ scalps at which the TMS was applied in three experimental conditions **(A–C)** and the stimulation schedule and protocols **(D,E)**.

This study adopted a randomized, placebo-controlled, and single-blind design. The participants were informed that they would receive one out of three TMS protocols without disclosing their details, such as possible effects or sensations felt. All participants completed the same preparation procedure before the TMS sessions. Both the real and sham TMS were delivered using the same machine. The TMS coils had a comparable outlook and emitted sounds. Group assignment, the delivery of the experimental and control protocols, and the test administration were performed by different research team members. These members did not communicate regarding the study. Depending on the group assignment, the participants completed five consecutive daily sessions of the iTBS, CTBS, or SHAM stimulation protocol (see Figure 1D). The participant sat upright on a comfortable chair with their head in a neutral position. The target brain areas on which the TMS was applied were located by the researcher. After the TMS, the participants completed two cognitive tasks at baseline and at the end of the 1st, 3rd, and 5th TMS sessions.

### 2.3 Stimulation protocols

The magnetic pulses were delivered by a Magstim Rapid^2^ stimulator (Magstim Limited, Whitland, United Kingdom) with a 70 mm diameter figure-of-8 coil. The 80% resting motor threshold (RMT) protocol for pulse intensity was adopted for all participants (Valchev et al., 2016).

The CTBS protocol was applied at the MCV6. The manual navigation method was used to locate the MCV6 because it is situated deep within the cerebellum behind the neck (Montreal Neurological Institute (MNI) coordinates [2.0, −68.6, −20.1]) (Wong et al., 2021). The manual method has been commonly used in other studies involving the cerebellum (e.g., Matsugi et al., 2019; Yao et al., 2022), and the location adopted was 1 cm inferior to the inion (Colnaghi et al., 2017b; Matsugi et al., 2019) (Figure 1A). The parameters for the CTBS were as follows: three-pulse bursts at 50 Hz delivered every 200 ms (5 Hz) (Rastogi et al., 2017) (Figure 1E) at 80% of the resting motor threshold. The total time of stimulation was 40 s (totaling 600 pulses).

The iTBS protocol was applied at the RpSMA. Different from MCV6, locating the RpSMA at the MNI coordinates [6, 20, 44] (Aron et al., 2007) was guided by the BrainSight^2^ navigation system (Rogue Research Inc.) (Figure 1B). The stimulation parameters for the iTBS were as follows: three-pulse bursts at 50 Hz every 200 ms in every 2 s train (Figure 1E). The 2 s train of pulses was repeated every 10 s for a total of 190 s (totaling 600 pulses) (Huang et al., 2008).

The SHAM protocol adopted the same parameters as those used for the CTBS but used a sham stimulation coil. The sham coil was placed at the vertex of the participant’s scalp (Figure 1C). The parameters for the SHAM condition were as follows: three-pulse bursts at 50 Hz delivered every 200 ms (5 Hz) (Figure 1E). The total stimulation time was 40 s (totaling 600 pulses). The sham coil did not generate any magnetic fields.

### 2.4 Behavioral test—processing speed tasks

**Figure 2:**
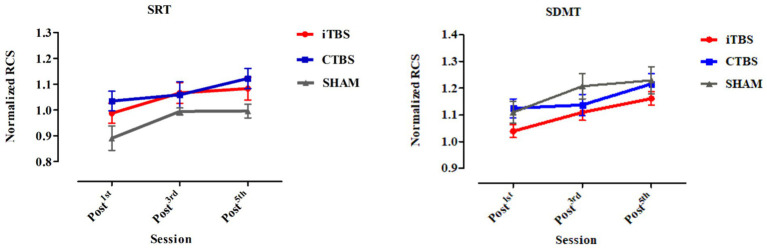
Normalized RCS on the SRT and SDMT of participants in the iTBS, CTBS, and SHAM groups on post-TMS1, post-TMS3, and post-TMS5 occasions. RCS, Rate-correct score; SRT, simple reaction time; SDMT, symbol digital modality test. The bars denote the means, and the error bars denote the SEs.

There were four test occasions: baseline, post-TMS1 (1st session), post-TMS3 (3rd session), and post-TMS5 (5th session) (Figure 2). The TMS2 and TMS4 tests were skipped to lower the possible over-practice effects due to the repeated testing among the participants. On each test occasion, participants were asked to complete two cognitive tasks. The two tests were custom-designed to tap into participants’ cognitive processing speeds in simple vs complex task-taking processes, i.e., simple reaction time (SRT) and modified SDMT, respectively (Supplementary appendices 1, 2). The tests were constructed with E-Prime 3.0 software (Psychology Software Tools, Inc.) and delivered online. The SRT was used in another study (Deary et al., 2007).

Simple reaction time (SRT): The participant was asked to place a finger on the “0” key; once the “0” appeared on the screen, the participant pressed the key as quickly as possible (Deary et al., 2007). There were 20 trials.

Symbol Digit Modality Test (SDMT): It loads onto attention, processing speed, and working memory (Leavitt, 2021). The electronic version was based on the modified version of SDMT operated on a touch-screen computer (Silva et al., 2018). The modified version was used in two other studies (Gawryluk et al., 2014; Manca et al., 2018). Nine symbol-digit pairs were shown on the upper part of the computer screen as the task keys. Below the symbol-digit pair keys presented a random symbol-digit pair as the stimulus of a trial. The participants were to indicate whether the presented pair would match with one of the nine keys above by pressing “1” (match) or “2” (not match) on the keyboard within 6 s. The symbol-digit pair stimuli were presented in a random order.

Both the reaction times and accuracy of the responses were recorded in each task. The participants were instructed to “complete the task as fast as they could.” There was no set time limit for the SRT, but 90 s time limit for the SDMT. Most of the participants completed the SRT in 16 s.

### 2.5 Data analyses

All analyses were conducted in accordance with the intent-to-treat (ITT) method (Xi et al., 2018; Andrade, 2022), and no data points were discarded. The participants’ demographic characteristics and their SRT and SDMT scores at the baseline were compared. The changes in the reaction times, i.e., the rate-corrected scores, in the SRT and SDMT (see below for the computation) were tested using the 3 × 3 repeated-measures analysis of covariance (RM-ANCOVA) with Group as the between-subject factor (iTBS, CTBS, and SHAM) and Time as the within-subject factor (post-TMS1, 3, and 5). The gender of the participants and their baseline test results were entered as the covariate as women were previously reported to have an advantage over men in processing speed (Siedlecki et al., 2019; Roivainen et al., 2021). Significant Group × Time effects were followed by *post-hoc* pairwise comparison with Bonferroni’s adjustment. Statistical significance for the comparisons was set at *p* = 0.001 (0.05/28) (two-tailed) after Bonferroni correction to the number of repeated measures. The software used for all tests was STATA version 17.0.

The rate-correct score (RCS) method (Liesefeld and Janczyk, 2019) was adopted to combine the RTs and accuracy rates for the SRT and SDMT of the participants. The RCS was derived for each participant who performed each of the two test tasks in the baseline and the three post-TMS occasions. The three post-TMS RCSs were normalized with the RCSs for the baseline to adjust for participants’ individual differences. The RCS can be interpreted as the number of correct responses per unit of time (Woltz and Was, 2006, p. 673) that addresses the potential speed-accuracy trade-offs with the following formula:


$$RCS_{i,j}=\frac{NC_{i,j}}{\sum_{K=1}^{n_{i,j}}RT_{i,j,k}}$$

where *NC_i,j_* is Participant *i*’s number of correct responses in Condition *j* and the denominator reflects the total time Participant *i* spent on trials in Condition *j* (in other words, the sum of RTs across all n*_ij_* trials of Participant *i* in Condition *j*) (Liesefeld and Janczyk, 2019).

We then applied structural equation modeling (SEM) to compare the effects of the iTBS and CTBS on the participants’ processing speed in the SRT and SDMT. Means and SDs of all variables used in the SEM were calculated and correlated with each other. The initial model, the iTBS vs SHAM model (M1), and the CTBS vs SHAM model (M2) were constructed. The data to model fit was evaluated using the Chi-squared (χ^2^), comparative fit index (CFI), and the root mean square error of approximation (RMSEA) (Schermelleh-Engel et al., 2003). The criteria set for a significant and good data to model fit were χ^2^/df < 2, CFI > 0.97, and RMSEA <0.05, and an acceptable fit by χ^2^/df < 3, CFI > 0.95, and RMSEA <0.08 (Schermelleh-Engel et al., 2003).

## 3 Results

### 3.1 Characteristics of participants

There were no significant differences in age, gender, years of education, or Mini-Mental State Examination scores in the participants among the iTBS, CTBS, and SHAM groups (Table 1). All participants completed the 5-day stimulation protocols and all the test tasks. Figure 2 shows the normalized RCS from the SRT and SDMT of participants in the three groups. RM-ANCOVA did not review significant Group × Time and Group effects on the normalized RCS of the SRT and SDMT (Table 2). The Time effect was statistically significant for the normalized RCS of the SRT [*F* (2, 65) = 9.34, η^2^ =  0.140, *p* < 0.001] and SDMT [*F* (2, 65) = 25.21, η^2^ = 0.306, *p* < 0.001]. The gender covariate was significant for the SRT [*F* (1, 65) = 6.28, η^2^ = 0.052, *p* = 0.014] but not for the SDMT [*F* (1, 65) = 1.50, η^2^ = 0.013, *p* = 0.223]. The baseline RCS covariate was not significant for the SRT [*F* (1, 65) = 0.52, η^2^ = 0.004, *p* = 0.472] and SDMT [*F* (1, 65) = 2.96, η^2^ = 0.025, *p* = 0.087].

**Table 1:** Demographic characteristics and MMSE scores of participants in the iTBS, CTBS, and SHAM groups.

	iTBS	CTBS	SHAM	χ^2^/*F*	*p*
	(*n* = 20)	(*n* = 20)	(*n* = 20)		
Age (Mean/SD)	23.8 (±2.67)	22.5 (±2.19)	23.0 (±1.97)	1.5	0.232
Sex (M/F)	7/13	6/14	7/13	0.15	0.928
Years of education (Mean/SD)	16.4 (±1.19)	16.1 (±1.47)	16.3 (±1.49)	0.34	0.715
MMSE (Mean/SD)	28.5 (±0.76)	28.25 (±1.33)	28.8 (±0.52)	1.73	0.186

MMSE, Mini-Mental State Examination; χ^2^/F is Mann–Whitney U-test and one-way ANOVA, respectively.

**Table 2:** The results of the Group, Time, and Group × Time effects on the normalized RCS of the participants on the SRT and SDMT.

Tasks	Groups	Group effects *p_a_*	Time effects *p_b_*	Interaction effect *p_c_*
SRT Normalized RCS	iTBS	0.241	<0.001***	0.669
	CTBS			
	SHAM			
SDMT Normalized RCS	iTBS	0.683	<0.001***	0.257
	CTBS			
	SHAM			

RCS, Rate-correct score; SRT, Simple reaction time; SDMT, Symbol digital modality test; ***p ≤ 0.001, **p ≤ 0.05. Bold values are measurement model.

### 3.2 The effects of stimulation on processing speed

#### 3.2.1 The initial SEM model

**Figure 3:**
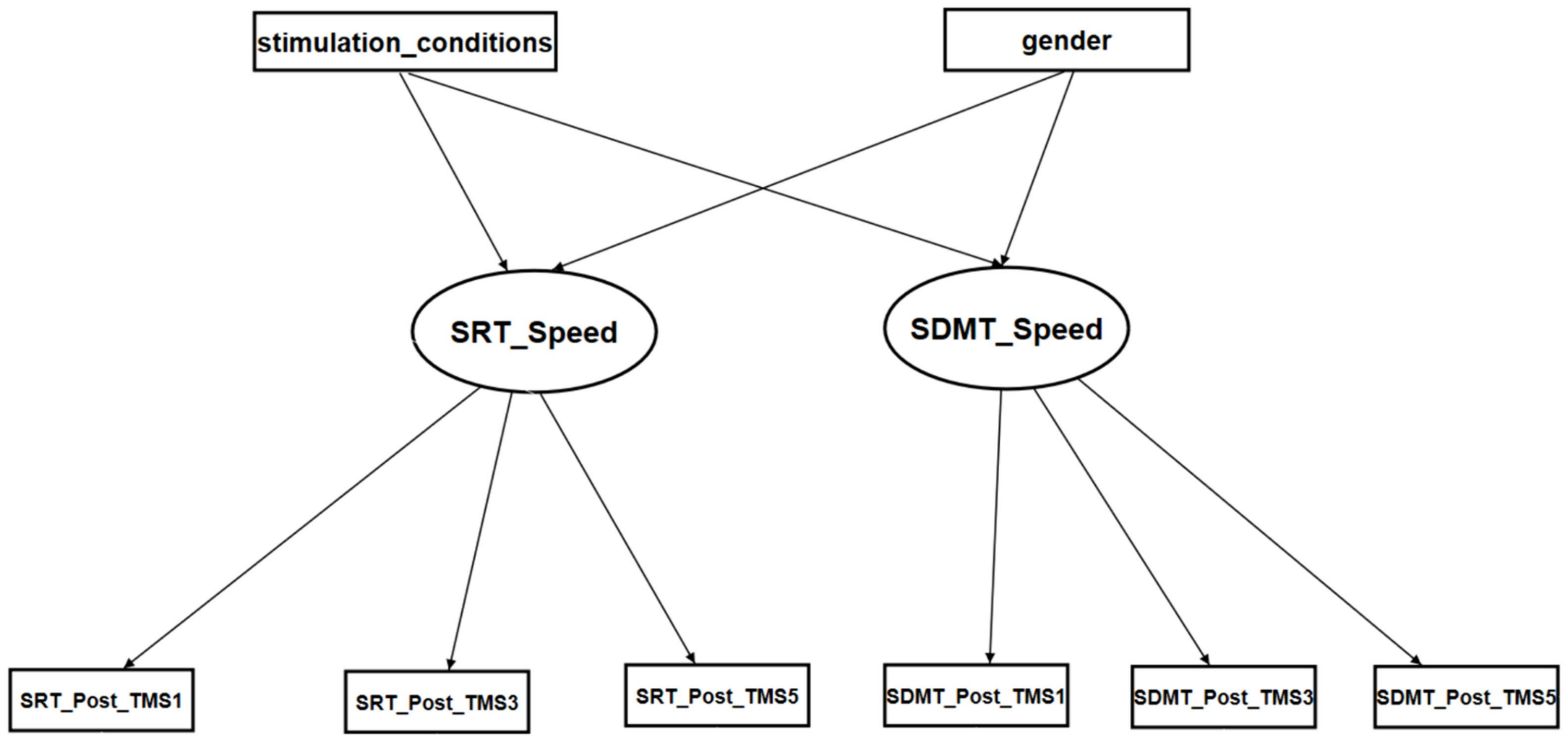
The initial SEM model for the reaction times of SRT and SDMT. Stimulation conditions: iTBS: 1, CTBS: 2, SHAM: 3. Gender: female: 0 and male: 1.

The initial model was constructed to include the TMS, gender of participants, and normalized RCS factors (Figure 3). The first-order factors were the iTBS, CTBS, and SHAM conditions, as well as gender. The second-order factor was the reaction times of the SRT and SDMT, which were affected by the first-order factors. The third-order factors were the normalized RCSs of the SRT and SDMT in the three test occasions, i.e., post-TMS1, post-TMS3, and post-TMS5.

#### 3.2.2 The iTBS (excitatory) vs SHAM effects on RpSMA

Supplementary appendix 3 summarizes the correlation patterns between the factor scores, mean scores, and standard deviation of the first-level and third-level factors. Furthermore, the “SRT_Post_TMS5” showed significant correlation with “SRT_Post_TMS3” (*r* = 0.657, *p* < 0.001), the “SDMT_Post_TMS3” showed significant correlation with “SDMT_Post_TMS1” and “SDMT_Post_TMS5” (*r* = 0.809, *p* < 0.001 and *r* = 0.877, *p* < 0.001, respectively), and the “SDMT_Post_TMS5” showed significant correlation with “SDMT_Post_TMS1” (*r* = 0.798, *p* < 0.001) (Supplementary appendix 3).

**Figure 4:**
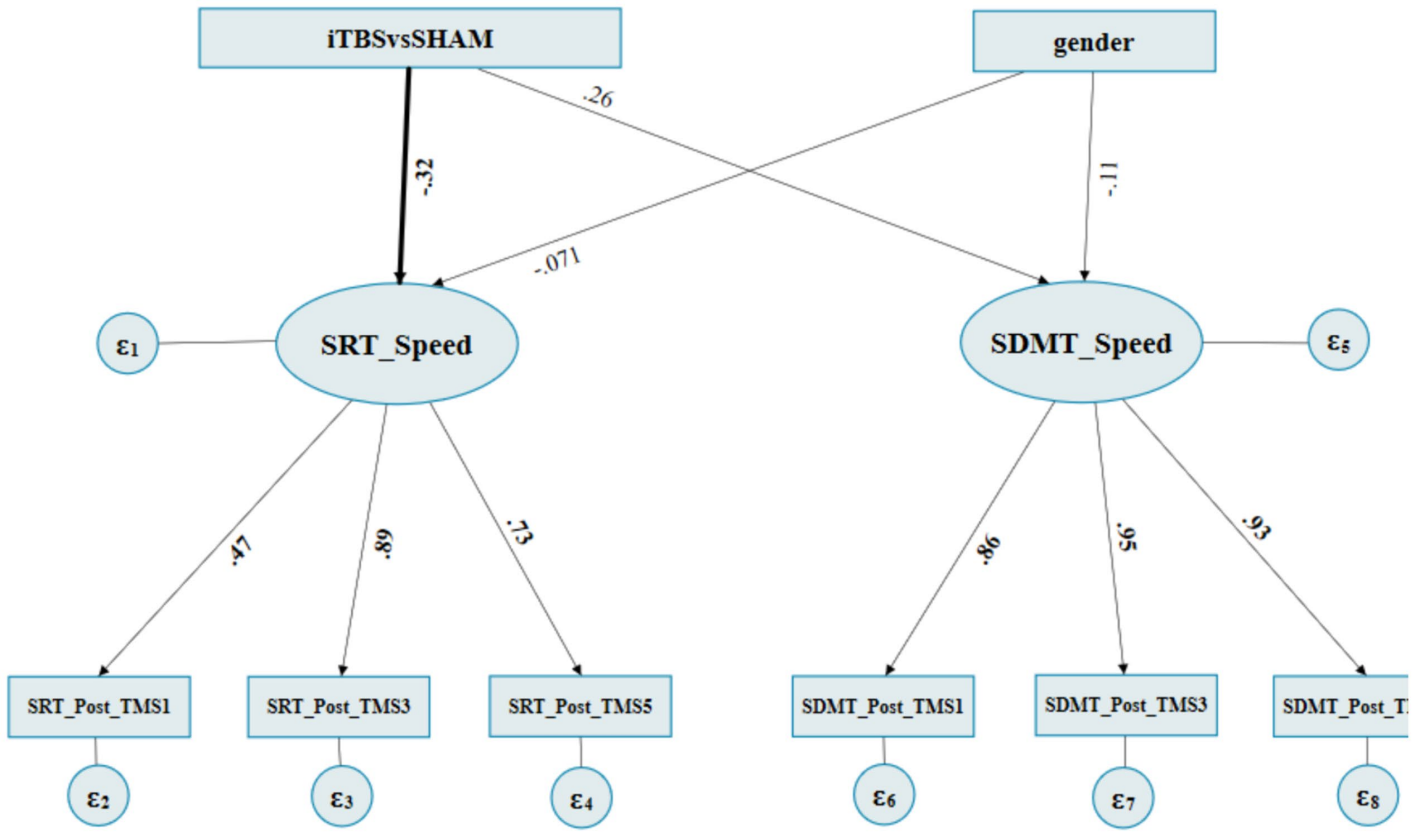
The final path diagram of SEM of iTBS vs SHAM on RpSMA. ITBS: 1, SHAM: 3. Gender: female: 0 and male: 1. SRT_Post_TMS1: SRT’s normalized RCSs at post^1st^ occasion, SRT_post_TMS3: SRT’s normalized RCSs at post^3rd^ occasion, SRT_Post_TMS5: SRT’s normalized RCSs at post^5th^ occasion. SDMT Post_TMS1: SDMT’s normalized RCSs at post^1st^ occasion, SDMT_post_TMS3: SDMT’s normalized RCSs at post^3rd^ occasion, SDMT_Post_TMS5: SDMT’s normalized RCSs at post^5th^ occasion.

Supplementary appendix 4 summarizes the iTBS vs SHAM on RpSMA (M1) fitness indices for the SEM. The estimate of the structural model indicated that the “iTBSvsSHAM” to the latent factor “SRT_speed” path was found to be significant; of which the effects were direct and negative (standardized regression coefficient = −0.320, *p* = 0.045) (Figure 4; Table 3). In contrast, the “iTBSvsSHAM” to the latent factor “SDMT_speed” path was not statistically significant (standardized regression coefficient = 0.258, *p* = 0.084). All the “gender” originated paths and effects were not significant.

**Table 3:** SEM regression weights of iTBS vs SHAM on RpSMA.

Measured variables	Standardized regression coefficient	S.E.	95% C.I.	*p*-value
**Estimate of structural model**				
iTBSvsSHAM→SRT_speed	−0.320	0.160	[−0.634, −0.007]	0.045**
iTBSvsSHAM→SDMT_speed	0.258	0.149	[−0.035, 0.551]	0.084
Gender→SRT_speed	−0.070	0.166	[−0.395, 0.254]	0.670
Gender→SDMT_speed	−0.105	0.155	[−0.409, 0.199]	0.498
**Loading of measurement model**				
SRT_speed→SRT_Post_TMS1	0.471	0.140	[0.196, 0.746]	0.001***
SRT_speed→SRT_Post_TMS3	0.891	0.119	[0.657, 1.126]	<0.001***
SRT_speed→SRT_Post_TMS5	0.732	0.117	[0.500, 0.962]	<0.001***
SDMT_speed→SDMT_Post_TMS1	0.856	0.048	[0.762, 0.951]	<0.001***
SDMT_speed→SDMT_Post_TMS3	0.946	0.031	[0.886, 1.007]	<0.001***
SDMT_speed→SDMT_Post_TMS5	0.926	0.034	[0.860, 0.993]	<0.001***

**p ≤ 0.05; ***p ≤ 0.001; S.E., standard error; 95% C.I., confidence interval. Bold values are measurement model.

The loading of the measurement model indicated that the latent factor “SRT_speed” to “SRT_Post_TMS1,” “SRT_Post_TMS3,” and “SRT_Post_TMS5” paths were all significant, with standardized factor loadings ranging from 0.471 to 0.891 (all *p* ≤ 0.001). All the other paths from the “SDMT_speed” to its subsequent SDMT reaction times were significant as well, which showed that the two latent variables “SRT_Speed” and “SDMT_Speed” can be well interpreted by three-level observative variables, respectively (Figure 4; Table 3).

#### 3.2.3 The CTBS (inhibitory) vs SHAM effects on the MCV6

Supplementary appendix 5 summarizes the correlation patterns between the factor scores, mean scores, and standard deviation of the first-level and third-level factors. Furthermore, the “SRT_Post_TMS5” showed significant correlation with “SRT_Post_TMS3” (*r* = 0.648, *p* < 0.001), the “SDMT_Post_TMS3” showed significant correlation with “SDMT_Post_TMS1” and “SDMT_Post_TMS5” (*r* = 0.766, *p* < 0.001 and *r* = 0.769, *p* < 0.001), and the “SDMT_Post_TMS5” showed significant correlation with “SDMT_Post_TMS1” (*r* = 0.776, *p* < 0.001) (Supplementary appendix 5).

**Figure 5:**
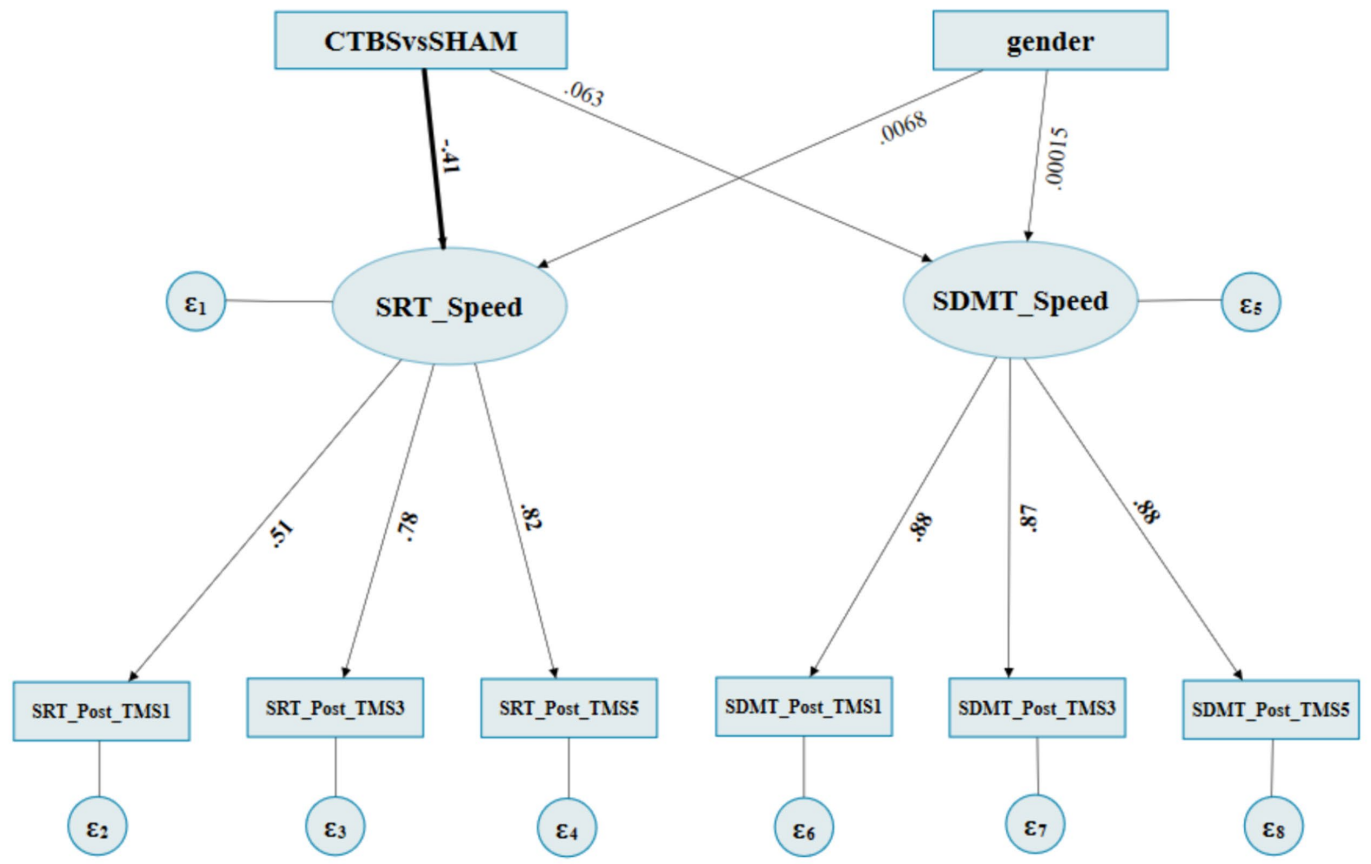
The final SEM path diagram of SEM of CTBS vs SHAM on MCV6. CTBS: 2, SHAM: 3. female: 0 and male: 1. SRT_Post_TMS1: SRT’s normalized RCSs at post^1st^ occasion, SRT_post_TMS3: SRT’s normalized RCSs at post^3rd^ occasion, SRT_Post_TMS5: SRT’s normalized RCSs at post^5th^ occasion. SDMT_Post_TMS1: SDMT’s normalized RCSs at post^1st^ occasion, SDMT_post_TMS3: SDMT’s normalized RCSs at post^3rd^ occasion, SDMT_Post_TMS5: SDMT’s normalized RCSs at post^5th^ occasion.

Supplementary appendix 6 summarizes the CTBS (inhibitory) vs SHAM on the MCV6 (M2) fitness indices for the SEM. The estimate of the structural model indicated that the “CTBSvsSHAM” to the “SRT_speed” latent factor path was found to be significant, of which the effects were direct and negative (standardized regression coefficient = −0.414, *p* = 0.007) (Figure 5; Table 4). In contrast, the “CTBSvsSHAM” to the “SDMT_speed” path was not statistically significant (standardized regression coefficient = 0.062, *p* = 0.708). All the “gender” originated paths and effects were not significant (Figure 5; Table 4).

**Table 4:** SEM regression weights of CTBS vs SHAM on MCV6.

Measured variables	Standardized regression coefficient	S.E.	95% C.I.	*p*-value
**Estimate of structural model**				
CTBSvsSHAM→SRT_speed	−0.414	0.154	[−0.716, −0.113]	0.007**
CTBSvsSHAM→SDMT_speed	0.062	0.167	[−0.265, 0.390]	0.708
Gender→SRT_speed	0.006	0.164	[−0.316, 0.330]	0.967
Gender→SDMT_speed	0.001	0.166	[−0.327, 0.327]	0.999
**Loading of measurement model**				
SRT_speed→SRT_Post_TMS1	0.509	0.149	[0.217, 0.801]	0.001***
SRT_speed→SRT_Post_TMS3	0.775	0.114	[0.551, 1.001]	<0.001***
SRT_speed→SRT_Post_TMS5	0.822	0.115	[0.595, 1.049]	<0.001***
SDMT_speed→SDMT_Post_TMS1	0.877	0.051	[0.776, 0.977]	<0.001***
SDMT_speed→SDMT_Post_TMS3	0.873	0.052	[0.771, 0.976]	<0.001***
SDMT_speed→SDMT_Post_TMS5	0.882	0.050	[0.784, 0.981]	<0.001***

**p ≤ 0.05; ***p ≤ 0.001; S.E., standard error; C.I., confident interval. Bold values are measurement model.

The loading of the measurement model indicated that the latent factor “SRT_speed” to the “SRT_Post_TMS1,” “SRT_Post_TMS3,” and “SRT_Post_TMS5” factor paths were also significant, with the standardized factor loadings ranging from 0.509 to 0.822 (all *p* ≤ 0.001). The “SDMT_speed” to its subsequent paths was significant as well, which showed that the two latent variables “SRT_Speed” and “SDMT_Speed” could be well interpreted by three-level observative variables, respectively (Figure 5; Table 4).

## 4 Discussion

This study aimed to test the roles of the RpSMA and MCV6 effective connectivity within the fronto-cerebellar network to subserve cognitive processing speed. The five-session TMS excitatory and inhibitory protocols were intended to initiate changes in the participants’ task-based reaction times. Despite the changes in the reaction times that had been observed across the stimulation sessions, they were at best regarded as marginal because they did not reach statistical significance. However, the results of structural equation modeling revealed systematic significant stimulation effects on both the RpSMA and MCV6, resulting in significant paths on the reaction time change factors. More importantly, the significant paths from the stimulation to the faster reaction time factors were only observed in the simple but not in the complex tasks. Excitation to the RpSMA relating to the faster reaction times in the simple task supports its “increase in the negative influence” role in the RpSMA➔MCV6 effective connectivity. Counterintuitively, inhibition of the MCV6 relating to the faster reaction times in the simple task did not support its unique role in the effective connective network to subserve processing speed. In other words, the iTBS protocol on the MCV6, which was supposed to reverse the “increase in the negative influence” originally received from the RpSMA, did not produce slower reaction times. The contradictory results obtained from the MCV6 are plausibly due to the influences exerted by the available short-range connective networks within the cerebellum.

Excitatory stimulation on the RpSMA did not produce significantly faster reaction times when compared with the sham condition. The non-significant results were yielded from conducting conventional Group × Time comparisons. Our findings are consistent with those reported in two previous studies, reporting the effects of excitatory stimulations on the RpSMA. One study employed quadripulse transcranial magnetic stimulation over the pre-SMA, which did not produce significant effects on participants’ performances in the simple choice reaction time task (Shimizu et al., 2020). Another study adopted a similar stimulation protocol to this study on the RpSMA, which showed significant modulation effects on the biceps brachii corticomotor excitability in individuals with tetraplegia (Mittal et al., 2022). However, several studies on similar topics involved excitatory stimulations applied to the dorsolateral prefrontal cortex. The results of these studies were largely equivocal on excitatory stimulations producing faster reaction times (Curtin et al., 2019; Song et al., 2020; Ngetich et al., 2022). Future studies can modify the stimulation protocols, such as increasing the dosage of the stimulation (intensity or duration) and enlarging the sample size. These strategies would increase the effect size of the stimulation, hence the chance of showing significant post-stimulation changes.

The results of the significant structural paths suggested the excitatory RpSMA effects related to faster post-stimulation reaction times in the simple, not complex task. The first level path was the excitatory RpSMA, which showed direct and negative effects on the latent simple task speed factor, while the second level path was the latent speed factor, which continued with similar relationships with the reaction times. The evidence renders support for Wong et al. (2021)’s proposition on the RpSMA “lowering negative influence” relationships with the MCV6 in the RpSMA➔MCV6 effective couple. The RpSMA or pre-SMA, part of the motor cortex, primarily relates to motor functions (motor inhibition, visuomotor sequence learning, the control of motor sequences, and modulation of the balancing of speed vs accuracy) (Georgiev et al., 2016; Obeso et al., 2017; Hanoğlu et al., 2020; Shimizu et al., 2020; Nakajima et al., 2022). One previous study reported that RpSMA subserved cognitive control such as response inhibition (Obeso et al., 2013). Our findings are contrary to those reported by Obeso et al., who showed that RpSMA was associated with simple rather than complex cognitive operations. The simple reaction time task employed in our study required the participants to decode symbols presented but make mono responses. The complex task was the symbol digit modalities task, requiring participants to decode, match, and respond according to the different symbol-digit pairs. In other words, the cognitive control processes would have been a part of the complex task but not part of the simple task. Future studies will need to replicate simple vs complex tasks to generate more robust results on the observed role of the RpSMA.

Similar to the RpSMA, inhibitory stimulation to the MCV6 did not produce significant changes in the participants’ reaction times. Deliberation on the significant direct and negative paths from the inhibitory MCV6 to the latent speed factor only in the simple but not in the complex task sheds light on its role in the RpSMA➔MCV6 effective connectivity. The MCV6-induced latent speed factor showed significant direct and negative paths to the simple task reaction times. In other words, the inhibitory MCV6 related to faster rather than slower reaction times, which was inconsistent with the study’s hypothesis. One plausible explanation for the contradictory results could be that the inhibitory stimulation was over-spilled to other cerebellar sites. Two studies reported inhibitory stimulations resulting in faster reaction times involving sites different from the MCV6. They were the stimulation at the 1–2 cm below the inion (adjacent to MCV6) (Heleven et al., 2021) or the right Crus I/Crus II (adjacent to MCV6) (Gatti et al., 2020). Both studies revealed significantly faster reaction times in complex tasks: picture sequencing (Heleven et al., 2021) or word pairing (Gatti et al., 2020). These studies further explained that inhibitory stimulation possibly would have modulated the inherent sequence processing, semantic function in language, or semantic memory function required in the complex tasks. The over-spilled inhibition speculation does not seem to offer insight into the counterintuitive findings. Another plausible line of explanation is from a recent study that applied inhibitory stimulation over the left cerebellum (1 cm below and 3 cm lateral to the inion), resulting in faster reaction times in a lexical decision task (Allen-Walker et al., 2018). Allen-Walker et al. attributed the faster reaction time to activate the automatic and fast feedback loops in the left cerebellar hemisphere after CTBS was applied with the left cerebellum or contralateral cerebral cortex (right temporal cortex) as a result of left cerebellar rTMS. An earlier study using the same lexical decision task and applying inhibitory stimulation to the site similar to MCV6 revealed deterioration of reaction times (Argyropoulos, 2011). Argyropoulos (2011) explained that the participants’ deteriorated performance could have been attributable to disrupted oculomotor processes by the inhibitory MCV6 essential for the reading during the task (Argyropoulos, 2011). Their argument was supported by a later study suggesting that the cerebellar stimulation could have modulated the primary motor cortex via the efferent path of the fronto-pontine-cerebello-thalamo-cortical loop (Grimaldi et al., 2014). However, the fronto-pontine-cerebello-thalamo-cortical loop is different from the fronto-cerebellar network, which contains the RpSMA➔MCV6 in this study. The latter is a long-range connectivity between the frontal cortex and the cerebellum without the dentate and motor thalamus (Wong et al., 2021). The lexical decision task involved semantic processing and higher cortical function, which is different from the simple reaction task in this study. Taken together, the inhibitory effects on the cerebellum are likely to vary with the location of stimulation and the content of the tasks. Future studies would explore the mechanism of combined existing connectivity or new models for the roles of the cerebellum in subserving cognitive processing speed.

A plausible explanation of the inhibitory MCV6 for the RpSMA➔MCV6 may be explained by the long- and short-range fronto-cerebellar effective connectivity revealed in our previous study (Wong et al., 2021). Long-range cerebellar connectivity plays a dominant role in subserving cognitive information processing (Deco et al., 2021). The RpSMA➔MCV6 couple is long-range connectivity. In the same study, Wong et al. revealed MCV6➔RCH6, a short-range connectivity, in which the MCV6 exerted a lower positive influence on the RCH6, predicting faster processing speed (Wong et al., 2021). Functional connectivity within the cerebellar networks was suggested to be associated with learning in young adults (Edde et al., 2020). The effect of RpSMA➔MCV6 on processing speed was found to be independent of that of MCV6➔RCH6 (Wong et al., 2021). We, therefore, speculate that the opposite effects manifested from the inhibitory MCV6 could have been intervened by the short-range cerebellar connectivity, such as the RCH6. However, the effect of short-range connectivity is outside the scope of this study. Further study should include both long- and short-range connectivities in building a comprehensive model for the cerebellum.

Another finding revealed in this study is stimulations at the RpSMA, and MCV6 showed effects on the simple but not complex tasks. These findings perhaps would have been confounded by the RpSMA➔MCV6 effective connectivity reported by Wong et al. (2021), which was based on the modified arrow test, which involved relatively simple cognitive operations. The simple reaction time task deployed in this study required basic information processes, such as encoding and discrimination of the figure “0,” and a one-to-one task rule of “press on a key” when seeing “0” (Deary et al., 2010). Our findings are consistent with an earlier study that a simple reaction time task involved activations of the premotor cortex, medial frontal gyrus, cerebellar vermis, and frontal–parietal cortex, which overlaps with the RpSMA and MCV6 (Kansaku et al., 2004). On the contrary, studies that employed more complex tasks, such as the SDMT deployed in this study, have been found to activate the other brain regions, including the frontal–parietal cortex, cingulate gyrus, and precuneus (Silva et al., 2018).

### 4.1 Limitations and future perspectives

There are a few limitations in this study. First, the simple task reaction time results are likely to be confounded by the modified arrow test, which involves relatively simple cognitive operations. Second, we adopted single and separate rather than multiple and simultaneous protocols over RpSMA and MCV6 for testing the RpSMA➔MCV6. The single and separate site stimulation could have weakened the effects modulating the fronto-cerebellar connective network. The non-significant modulating effects for the complex task performance could have been due to the five-session protocols producing inadequate stimulation effects or the ceiling effect among the young, healthy participants. The task choices in our study were only limited to simple and complex tasks in their relativity, which do not represent the wide spectrum of cognitive operations. Finally, the study’s sample size was relatively small, which could have weakened the power to detect any possibly significant changes, particularly in the complex task’s performances. Future studies should replicate the study with a large sample size and adopt more stimulation conditions, such as dual-site simultaneous protocol, increasing the dosage of the stimulation, and more cognitive tasks with different mental operations. The validity of the stimulation to modulate the fronto-cerebellar network would need to go beyond a behavioral study, such as a brain imaging method, to quantify the post-stimulation functional changes within the network. The young, healthy participants of this study limit the generalization of the findings to other healthy age groups and diagnostic groups. Readers should interpret the results with caution.

## 5 Conclusion

We attempted to understand the role of the individual neural substrates in the RpSMA➔MCV6 effective connective couple within the fronto-cerebellar network. The hypotheses set for the study were partly supported. Based on the structural equation modeling results, excitatory stimulation at the RpSMA showed significant paths to faster reaction times in the simple cognitive task. The results supported the RpSMA playing a lower negative effect role, as proposed in our previous study inhibiting the MCV6, which subserves faster processing speed. However, the results from the inhibitory MCV6 showed that significant paths to the faster reaction times in the simple cognitive tasks did not support the hypothesis. The anticipated inhibition of the MCV6, which was supposed to be associated with slower reaction times, could have been compromised by the short-range cerebellar connectivity, such as the RCH6.

## Data availability statement

The raw data supporting the conclusions of this article will be made available by the authors, without undue reservation.

## Ethics statement

The studies involving humans were approved by the Ethics Committee of the Rehabilitation Hospital affiliated with the Fujian University of Traditional Chinese Medicine. The studies were conducted in accordance with the local legislation and institutional requirements. The participants provided their written informed consent to participate in this study.

## Author contributions

NZ: Data curation, Formal analysis, Investigation, Methodology, Software, Writing – original draft, Writing – review & editing, Visualization. JT: Project administration, Software, Supervision, Writing – review & editing, Investigation. CW: Methodology, Software, Writing – review & editing. JW: Methodology, Software, Writing – review & editing. JL: Methodology, Software, Writing – review & editing. LC: Conceptualization, Project administration, Supervision, Writing – original draft, Writing – review & editing, Methodology. TL: Conceptualization, Methodology, Writing – original draft, Writing – review & editing. YX: Writing – review & editing. CC: Conceptualization, Funding acquisition, Investigation, Methodology, Project administration, Supervision, Writing – original draft, Writing – review & editing.

## Supplementary material

SUPPLEMENTARY APPENDIX 1 Task design of simple reaction time task (SRT).

SUPPLEMENTARY APPENDIX 2 Task design of SDMT.
