# Supplementary figures and images for: Theta burst stimulation on the fronto-cerebellar connective network promotes cognitive processing speed in the simple cognitive task
Source: Front Hum Neurosci. 2024 Jul 19;18:1387299. doi: 10.3389/fnhum.2024.1387299 (PMC11417469; doi:10.3389/fnhum.2024.1387299)

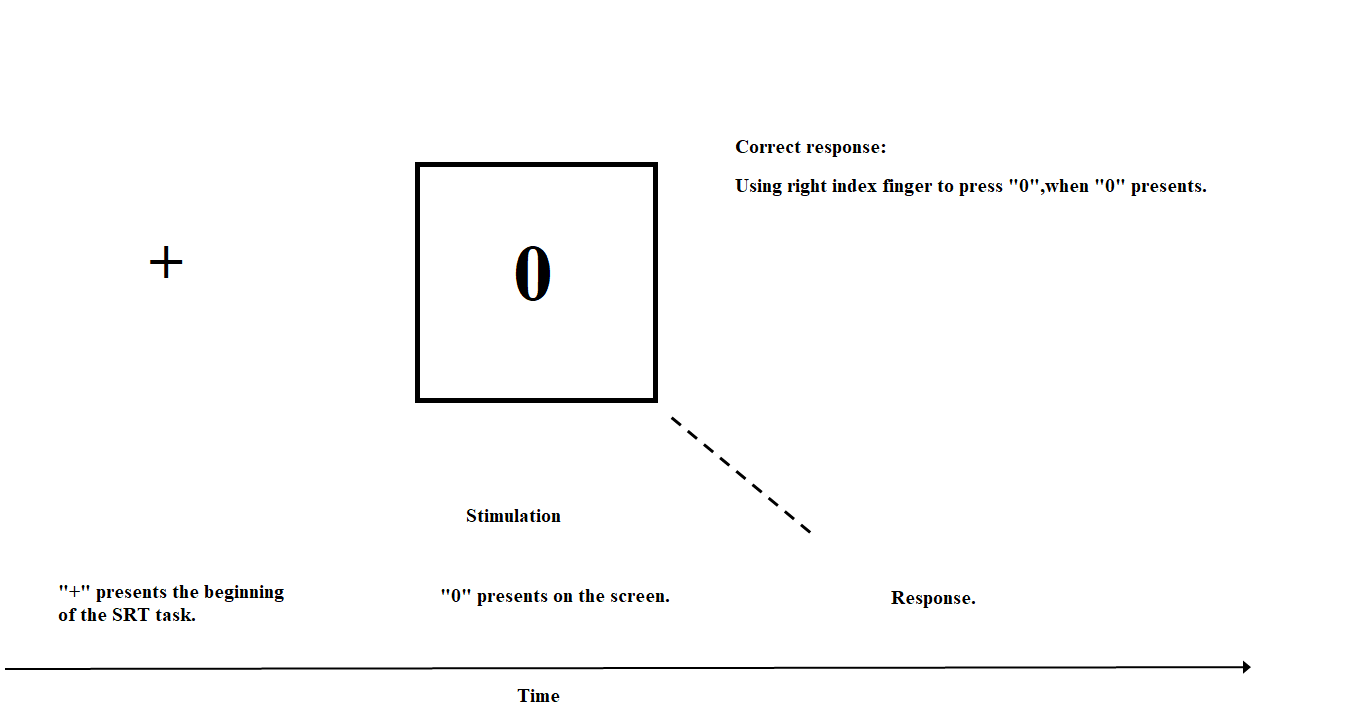

Supplement: SUPPLEMENTARY APPENDIX 1 — Task design of simple reaction time task (SRT). [file Image_1.TIF]

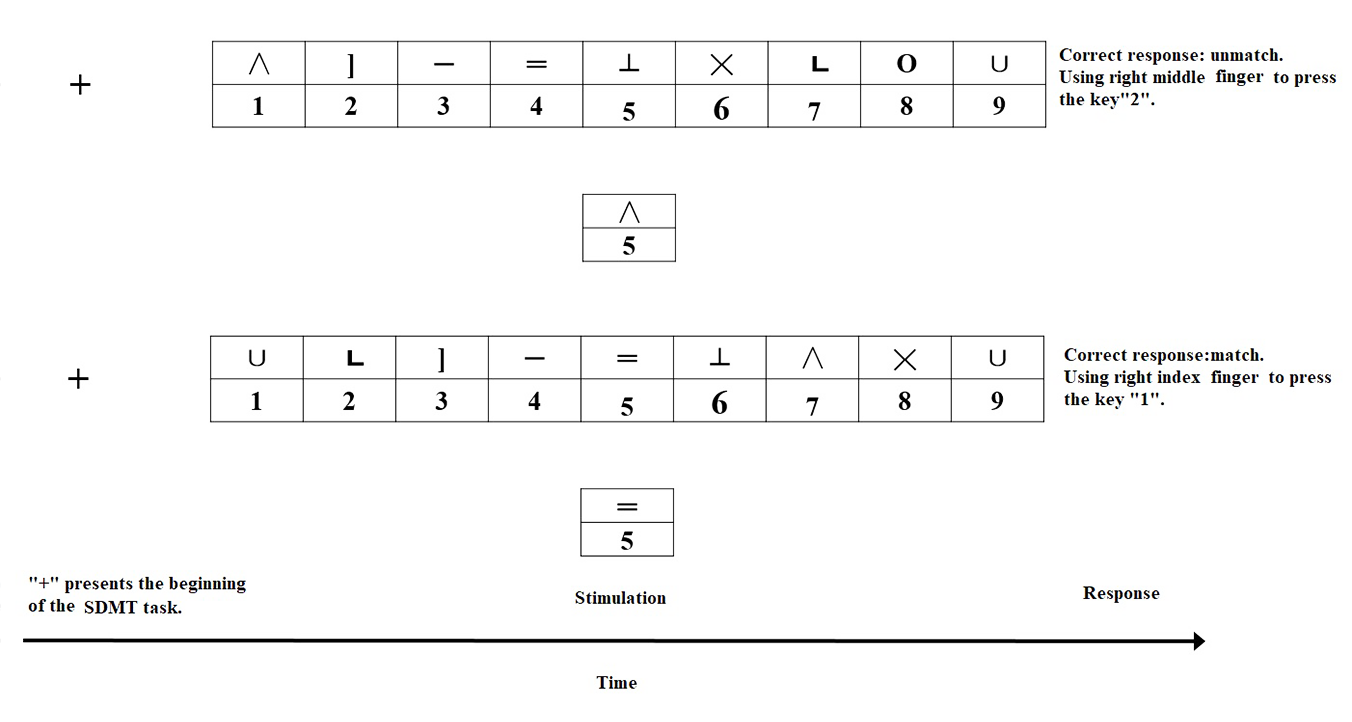

Supplement: SUPPLEMENTARY APPENDIX 2 — Task design of SDMT. [file Image_2.TIF]
