# Supplementary material for: Theta burst stimulation on the fronto-cerebellar connective network promotes cognitive processing speed in the simple cognitive task
Source: Front Hum Neurosci. 2024 Jul 19;18:1387299. doi: 10.3389/fnhum.2024.1387299 (PMC11417469; doi:10.3389/fnhum.2024.1387299)
Supplement: Supplementary file 3 [file Table_1.DOCX]

**Appendix 3**. Correlation among variables used in iTBS versus SHAM on RpSMA (M1).

| M1 | 1 | 2 | 3 | 4 | 5 | 6 | 7 | 8 |
| --- | --- | --- | --- | --- | --- | --- | --- | --- |
| 1 iTBSvsSHAM(1,iTBS.3,SHAM) | - |  |  |  |  |  |  |  |
| 2 gender(0,female.1,male) | -0.000 | - |  |  |  |  |  |  |
| 3 SRT_Post_TMS1 | -0.248 | -0.221 | - |  |  |  |  |  |
| 4 SRT_Post_TMS3 | -0.261 | -0.043 | 0.426 | - |  |  |  |  |
| 5 SRT_Post_TMS5 | -0.264 | -0.035 | 0.298 | **0.657***** | - |  |  |  |
| 6 SDMT_Post_TMS1 | 0.235 | 0.003 | 0.051 | 0.002 | 0.011 | - |  |  |
| 7 SDMT_Post_TMS3 | 0.272 | -0.108 | -0.081 | 0.009 | 0.079 | **0.809***** | - |  |
| 8 SDMT_Post_TMS5 | 0.195 | -0.132 | -0.028 | 0.117 | 0.142 | **0.798***** | **0.877***** | - |
| Mean | 1 | 0.35 | 0.988 | 1.066 | 1.083 | 1.039 | 1.109 | 1.161 |
|  | 3 | 0.35 | 0.891 | 0.995 | 0.996 | 1.110 | 1.208 | 1.229 |
| SD | 0.00 | 0.489 | 0.175 | 0.181 | 0.198 | 0.109 | 0.135 | 0.115 |
|  | 0.00 | 0.489 | 0.211 | 0.061 | 0.118 | 0.183 | 0.213 | 0.223 |

Note: ****p* ≤ 0.001.
