# Supplementary material for: Theta burst stimulation on the fronto-cerebellar connective network promotes cognitive processing speed in the simple cognitive task
Source: Front Hum Neurosci. 2024 Jul 19;18:1387299. doi: 10.3389/fnhum.2024.1387299 (PMC11417469; doi:10.3389/fnhum.2024.1387299)
Supplement: Supplementary file 5 [file Table_3.DOCX]

**Appendix 5**. Correlation among variables used in CTBS versus SHAM on RpSMA (M2).

| M2 | 1 | 2 | 3 | 4 | 5 | 6 | 7 | 8 |
| --- | --- | --- | --- | --- | --- | --- | --- | --- |
| 1 CTBSvsSHAM(2,CTBS.3,SHAM) | - |  |  |  |  |  |  |  |
| 2 gender(0,female.1,male) | 0.053 | - |  |  |  |  |  |  |
| 3 SRT_Post_TMS1 | -0.359 | -0.049 | - |  |  |  |  |  |
| 4 SRT_Post_TMS3 | -0.194 | -0.014 | 0.449 | - |  |  |  |  |
| 5 SRT_Post_TMS5 | -0.397 | 0.001 | 0.348 | **0.648***** | - |  |  |  |
| 6 SDMT_Post_TMS1 | -0.044 | -0.051 | 0.211 | 0.033 | 0.129 | - |  |  |
| 7 SDMT_Post_TMS3 | 0.180 | 0.057 | 0.109 | -0.028 | -0.006 | **0.766***** | - |  |
| 8 SDMT_Post_TMS5 | 0.034 | -0.092 | 0.029 | -0.003 | 0.066 | **0.776***** | **0.769***** | - |
| Mean | 2 | 0.3 | 1.034 | 1.059 | 1.122 | 1.125 | 1.137 | 1.216 |
|  | 3 | 0.35 | 0.890 | 0.994 | 0.995 | 1.110 | 1.208 | 1.229 |
| SD | 0 | 0.470 | 0.170 | 0.227 | 0.174 | 0.160 | 0.178 | 0.171 |
|  | 0 | 0.489 | 0.211 | 0.061 | 0.119 | 0.183 | 0.213 | 0.223 |

Note: ****p* ≤ 0.001, bonferroni’s correction.
