# Supplementary material for: Theta burst stimulation on the fronto-cerebellar connective network promotes cognitive processing speed in the simple cognitive task
Source: Front Hum Neurosci. 2024 Jul 19;18:1387299. doi: 10.3389/fnhum.2024.1387299 (PMC11417469; doi:10.3389/fnhum.2024.1387299)
Supplement: Supplementary file 6 [file Table_4.DOCX]

**Appendix6**. Summary of CTBS versus SHAM on MCV6 (M2) SEM fitness indices.

| Fitness index | M2 | Recommended  good values | Recommended  acceptable values |
| --- | --- | --- | --- |
| *χ^2^* | 19.760 | - | - |
| *df* | 17 | - | - |
| *χ^2^*/df | 1.162 | < 2 | < 3 |
| RMSEA | 0.065 | < 0.05 | < 0.08 |
| CFI | 0.975 | > 0.90 | > 0.95 |
| TLI | 0.960 | > 0.90 | > 0.95 |
| SRMR | 0.073 | < 0.05 | < 0.08 |

Note: *χ*2, chi-square; *df*, degree of freedom; RMSEA, Root-mean square error of approximation; CFI, Comparative fit index; TLI, Tucker-Lewis index; SRMR, Standardized root mean square residual.
